# Carboxylated branched poly(β-amino ester) nanoparticles enable robust cytosolic protein delivery and CRISPR-Cas9 gene editing

**DOI:** 10.1126/sciadv.aay3255

**Published:** 2019-12-06

**Authors:** Yuan Rui, David R. Wilson, John Choi, Mahita Varanasi, Katie Sanders, Johan Karlsson, Michael Lim, Jordan J. Green

**Affiliations:** 1 Department of Biomedical Engineering, Institute for NanoBioTechnology, and Translational Tissue Engineering Center, Johns Hopkins University School of Medicine, Baltimore, MD, USA.; 2 Department of Neurosurgery, Johns Hopkins University School of Medicine, Baltimore, MD, USA.; 3 Department of Oncology, Johns Hopkins University School of Medicine, Baltimore, MD, USA.; 4 Departments of Ophthalmology, Materials Science and Engineering, and Chemical and Biomolecular Engineering, and the Bloomberg~Kimmel Institute for Cancer Immunotherapy, Johns Hopkins University School of Medicine, Baltimore, MD, USA.

## Abstract

Efficient cytosolic protein delivery is necessary to fully realize the potential of protein therapeutics. Current methods of protein delivery often suffer from low serum tolerance and limited in vivo efficacy. Here, we report the synthesis and validation of a previously unreported class of carboxylated branched poly(β-amino ester)s that can self-assemble into nanoparticles for efficient intracellular delivery of a variety of different proteins. In vitro, nanoparticles enabled rapid cellular uptake, efficient endosomal escape, and functional cytosolic protein release into cells in media containing 10% serum. Moreover, nanoparticles encapsulating CRISPR-Cas9 ribonucleoproteins (RNPs) induced robust levels of gene knock-in (4%) and gene knockout (>75%) in several cell types. A single intracranial administration of nanoparticles delivering a low RNP dose (3.5 pmol) induced robust gene editing in mice bearing engineered orthotopic murine glioma tumors. This self-assembled polymeric nanocarrier system enables a versatile protein delivery and gene editing platform for biological research and therapeutic applications.

## Teaser

New biodegradable nanoparticles shuttle proteins inside cells and enable robust gene editing.

## INTRODUCTION

Since the introduction of the first recombinant protein drug—human insulin (*1*)—in 1982, the number of therapeutic proteins and the frequency of their use have markedly increased. These diverse and dynamic macromolecules have been used to treat diseases ranging from metabolic disorders to cancer (*2*) and are important in applications such as genome editing (*3*) and synthetic biology (*4*). However, their high molecular weight and overall hydrophilicity render most proteins essentially membrane impermeable (*5*), limiting most current protein therapeutics to extracellular targets. As proteins have the potential to target intracellular pathways with high specificity and fewer side effects (*6*), it is imperative to develop novel strategies for efficient, functional, and cytosolic protein delivery.

Cytosolic protein delivery vehicles must overcome several barriers such as cargo encapsulation, cellular internalization, escape from endo-lysosomes, and cytosolic cargo release (*7*). One well-characterized approach is the covalent modification of the protein of interest with protein transduction domains such as the TAT protein from HIV (*8*). This strategy has been shown to enable rapid cellular internalization of a wide variety of proteins but requires chemical modifications that could alter the bioactivity of the native protein. More recently, several studies have reported the use of self-assembled protein delivery vehicles based on lipid-like (*9*), polymeric (*10*), or hybrid materials (*11*–*13*). These methods still face limitations such as the need for purification steps, low protein loading efficiency, and limited applicability to certain cargo types, prompting the need for improved self-assembled protein delivery systems.

Hyperbranched cationic poly(β-amino ester)s (PBAEs) have recently generated interest as an efficient gene delivery material for highly negatively charged nucleic acids (*14*–*16*). These amphiphilic, pH-sensitive polymers are synthesized via facile Michael addition reactions and have been shown to have robust transfection capabilities under challenging conditions as well as efficient endosomal escape properties. However, cationic polymers, such as PBAEs, form self-assembled nucleic acid nanoparticles mainly through electrostatic interactions, which are generally insufficient to encapsulate proteins of diverse surface charge.

In this study, we synthesized and validated a new biomaterial class of hyperbranched PBAE containing both cationic and anionic charges. This was accomplished through polymer end-capping with carboxylate ligands derived from amino acid–like precursors. Polymers were assembled into nanoparticles with proteins by simple mixing in aqueous buffer. We hypothesized that the carboxylate ligands can enhance polymer-protein interactions for nanoparticle assembly via increased hydrogen bonding and hydrophobic effects in addition to electrostatic interactions. Furthermore, we found that differential polymer end-group hydrophobicity affected protein complexation capabilities as well as nanoparticle internalization and endosomal escape. Our delivery platform enabled functional cytosolic delivery of proteins ranging from 27 to 160 kDa in molecular weight with varying surface charges. Encapsulation of Cas9 ribonucleoproteins (RNPs) enabled efficient gene editing in vitro and in vivo, further highlighting the robustness and therapeutic utility of these nanocarriers.

## RESULTS

### Polymer synthesis and screening

**Fig. 1:**
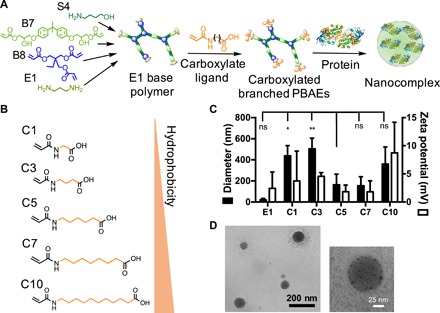
Design and characterization of self-assembled carboxylated branched PBAE protein nanoparticles. (**A**) Assembly of carboxylated branched PBAEs with proteins. (**B**) Structures of carboxylate ligands C1 to C10, arranged in order of increasing hydrophobicity. (**C**) Hydrodynamic diameter and zeta potentials of nanoparticles formulated with BSA (30 w/w) as measured by DLS. Data are presented as means + SD (*n* = 3). Statistical comparisons of nanoparticle diameter were performed with one-way analysis of variance (ANOVA) with Dunnett’s post hoc tests against the C5 group. *P < 0.05 and **P < 0.01. ns, not significant. Similar statistical comparisons were made with zeta potential data, and no significant differences were observed. (**D**) Representative transmission electron microscopy (TEM) images of C5/BSA nanoparticles.

Hyperbranched PBAEs were synthesized via a stepwise copolymerization reaction between acrylate-containing monomers bisphenol A glycerolate (1 glycerol/phenol) diacrylate (B7) and trimethylolpropane triacrylate (B8) and amino alcohol monomer 4-amino-1-butanol (S4). B monomers were added in molar excess to yield acrylate-terminated polymers, which were end-capped with the diamine-containing small-molecule 1,3-diaminopropane (E1) to yield E1 base polymers (Fig. 1A). These polymers underwent a second round of end-capping reactions with carboxylate ligands (fig. S1), which were synthesized via reaction of a series of amino acid–like precursors with acryloyl chloride. Ligands were named according to the number of carbon atoms between the amide and carboxylic acid groups, with C1 deriving from the amino acid glycine (fig. S2). Five carboxylate ligands ranging from C1 to C10 were synthesized to investigate the effect of end-cap hydrophobicity on the protein encapsulation and delivery capabilities of the polymers. We hypothesized that the combination of the biodegradable and hyperbranched polymer backbone, which has been shown to be amphiphilic and pH sensitive, (*15*) and carboxylate end-capping ligands, capable of forming hydrogen bonds and salt bridges with proteins, would result in a versatile protein delivery polymer platform.

**Fig. 2:**
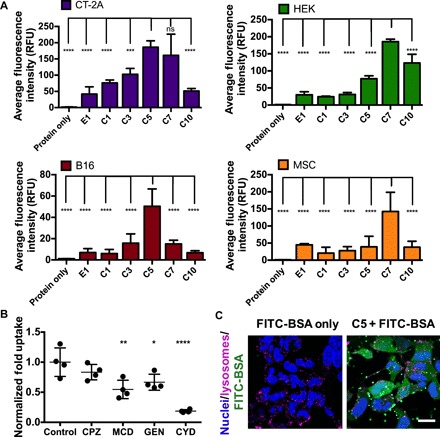
Carboxylated PBAE nanoparticles mediate cytosolic protein delivery. (**A**) Average fluorescence intensity of cells treated with carboxylated PBAE nanoparticles encapsulating FITC-BSA (300 ng of FITC-BSA per well, 20 w/w). Data are presented as means + SD (*n* = 4); statistical significance is determined by one-way ANOVA with Dunnett’s post hoc tests comparing uptake levels to that of the nanoparticle formulation achieving the highest levels of FITC-BSA uptake in each cell line. ***P < 0.001 and ****P < 0.0001. (**B**) Uptake by HEK cells in the presence of different endocytosis inhibitors. CPZ, chlorpromazine; MCD, methyl-β-cyclodextrin; GEN, genistein; CYD, cytochalasin D. Data are presented as means ± SD; statistical significance is determined by one-way ANOVA with Dunnett’s post hoc tests as compared to the control group (*n* = 4). *P < 0.05, **P < 0.01, and ****P < 0.0001. (**C**) Confocal images of HEK cells treated with C5/FITC-BSA nanoparticles or protein alone for 4 hours. Scale bar, 10 μm.

To investigate the protein encapsulation capabilities of the polymers, we formulated self-assembled polymeric nanoparticles with bovine serum albumin (BSA). At a polymer-protein weight ratio (w/w) of 30, all carboxylate-terminated polymers in the series formed nanoparticles ranging from 200 to 500 nm in hydrodynamic diameter with surface charges close to neutral (Fig. 1C), whereas the E1-terminated polymer, useful for self-assembly with nucleic acids (*17*), failed to effectively form nanoparticles with BSA. The diameter of the nanoparticles formulated from carboxylated polymers had a biphasic response dependent on the number of carbon atoms between the amide and carboxylic acid groups. Polymers end-capped with ligands C5 and C7 formed the smallest nanoparticles, and polymers with lower or higher end-cap carbon length formed much larger nanoparticles. Moreover, the same biphasic response was observed functionally when these polymers were used to deliver fluorescein isothiocyanate (FITC)–labeled BSA intracellularly (Fig. 2A). In all four cell lines evaluated [CT-2A murine glioma, human embryonic kidney (HEK)–293T, B16-F10 murine melanoma, and MSC-083 primary human adipose-derived mesenchymal stem cells], polymer surface engineered with C5 or C7 moieties enabled the highest levels of intracellular nanoparticle–mediated protein uptake. These data indicate that end-cap hydrophobicity and the spacing length between charges play a major role in the interactions between polymer and protein during nanoparticle self-assembly, which, in turn, affects interactions between nanoparticles and cells during cellular uptake. The high levels of protein nanoparticle uptake did not result in notable levels of cytotoxicity, and the viability of cells treated with nanoparticles was >70% for all polymers and cell lines tested when polymers were used at standard conditions (<0.15 mg/ml) (fig. S3).

When nanoparticle internalization pathways were probed by selectively inhibiting endocytosis pathways using small-molecule drugs, we found that pretreatment with cytochalasin D decreased nanoparticle uptake by over 80%, suggesting that nanoparticles were internalized primarily by macropinocytosis (Fig. 2B). Methyl-β-cyclodextrin and genistein also significantly decreased cellular uptake while chlorpromazine had negligible effects, indicating that nanoparticles were also taken up through lipid raft– and caveolin-mediated endocytosis but not through clathrin-mediated endocytosis. Last, confocal laser scanning microscopy images of cells after 4-hour incubation with C5/FITC-BSA nanoparticles revealed diffuse FITC-BSA signal throughout the cytosol, indicating that nanoparticles successfully escaped degradative endo-lysosomes to enable cytosolic protein delivery (Fig. 2C and fig. S4).

### Endosomal disruption characterization via Gal8-GFP recruitment assay

**Fig. 3:**
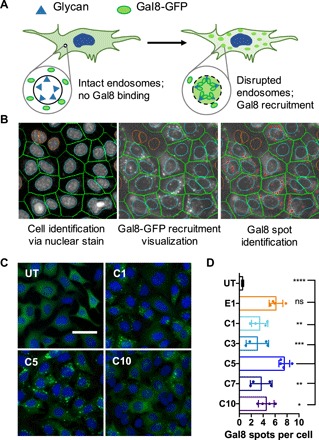
Gal8-GFP recruitment assay to assess nanoparticle-mediated endosomal disruption. (**A**) Gal8 recruitment overview. In cells with intact endosomes, Gal8-GFP is dispersed throughout endosomes with no interactions with intraendosomal glycans. Gal8-GFP binds glycans in disrupted endosomes, resulting in punctate fluorescent dots. (**B**) Gal8-GFP recruitment was quantified by image-based analysis. Individual cells were identified through nuclear staining (left); Gal8-GFP recruitment could be visualized in the green fluorescence channel (middle); punctate GFP^+^ spots were identified and counted (red dots). (**C**) Representative images of Gal8-GFP^+^ B16 cells treated with carboxylated PBAE/BSA nanoparticles (125-ng BSA per well, 25 w/w; scale bar, 50 μm). (**D**) Endosomal disruption level quantified by the number of Gal8-GFP spots per cell. Data are presented as means ± SD; statistical significance is determined by one-way ANOVA with Dunnett’s post hoc tests as compared to the C5 group (*n* = 4). *P < 0.05, **P < 0.01, ***P < 0.001, and ****P < 0.0001.

We further characterized the endosomal escape capabilities of carboxylated branched PBAE nanoparticles using an assay based on the recruitment of galectin 8 (Gal8) to disrupted endosomal membranes similar to the method recently innovated by Kilchrist *et al*. (*18*). Gal8 is a cytosolic protein that binds to glycosylation moieties located selectively on the inner leaflets of endosomal membranes. Using a PiggyBac transposon, we created a cell line stably expressing a Gal8-GFP (green fluorescent protein) fusion protein. Endosomal rupture exposes Gal8-binding sites to cytosolic Gal8-GFP, and Gal8-GFP recruitment results in punctate fluorescent spots at disrupted endosomes (Fig. 3A). After staining with Hoechst 33342 nuclear dye to allow for cell identification, automated high-content imaging analysis can then be used to identify punctate Gal8-GFP spots and calculate the number of Gal8-GFP spots per cell as an indicator of the level of nanoparticle-mediated endosomal disruption (Fig. 3B).

Our results revealed that among the carboxylate end-capped polymers, polymer C5 enabled the highest level of endosomal disruption (Fig. 3D). This was not due to the buffering capabilities of these polymers, as pH titration experiments showed that there was no significant difference in buffering capacity among the different carboxylated polymers (fig. S5A). It is also important to note that there was no significant difference between the Gal8-GFP recruitment levels of nanoparticles formed with the E1 base polymer and those formed with polymer C5. Polymer end-capping with carboxylate ligands of shorter chain lengths (e.g., C1 and C3) resulted in a decrease in endosomal disruption levels. This may be explained by the fact that the E1 monomer itself interacts with endosomal membranes in a way that causes disruption, as was demonstrated in previous reports using this molecule as an end cap to efficiently deliver plasmid DNA (*17*). Further end-capping with carboxylate ligands masked this effect, and endosomal disruption became dependent on hydrophobic chain length. Certain PBAEs have also been shown to form polymer-only, micellar nanoparticles in the absence of nucleic acid or protein cargo due to their amphiphilic structure (*19*). Thus, the paradoxical low FITC-BSA uptake and high Gal8-GFP recruitment observed in E1 nanoparticles could be explained by E1 base polymers not adequately forming protein-encapsulated nanoparticles and mainly forming polymer-only nanoparticles, which caused endosomal disruption after endocytosis. Together, our data indicate that polymer C5 outperformed all other polymers in the series and was chosen for use in all subsequent experiments.

### Robustness of C5/PBAE nanoparticles

**Fig. 4:**
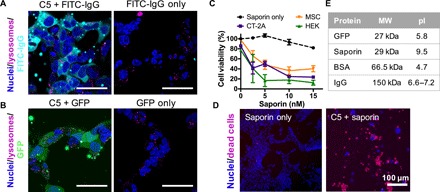
Carboxylated C5 polymeric nanoparticles for cytosolic delivery of different protein types. (**A** and **B**) Confocal images of HEK cells treated with C5 nanoparticles encapsulating FITC-IgG (A) and GFP (B) for 4 hours; 450 ng of protein was delivered per well at 30 w/w (scale bars, 50 μm). (**C**) Functional delivery of ribosome-inactivating protein saporin resulted in significant levels of cell death; the final polymer concentration per well was 0.075 μg/μl. Data are presented as means ± SD (*n* = 4). (**D**) Representative images of CT-2A cells treated with 10 nM naked saporin or C5/saporin nanoparticles. (**E**) Molecular weight (MW) and isoelectric point (pI) of proteins delivered by C5 nanoparticles.

We further examined the robustness of C5 end-capped polymers by using them for cytosolic delivery of a variety of proteins. C5 polymers successfully encapsulated FITC-labeled human immunoglobulin G (IgG) (FITC-IgG) and GFP, respectively, and enabled diffuse cytosolic delivery of both (Fig. 4). To investigate the capability for functional protein delivery, we further used C5 polymers to encapsulate the ribosome-inactivating protein saporin, a potent toxin lacking cellular internalization domains (Fig. 4C) (*20*). In all three cell lines tested, C5/saporin nanoparticles induced high levels of cell death even at very low saporin doses [EC_50_ (median effective concentration) < 5 nM]. This indicates that C5 nanoparticles enabled functionally intact saporin proteins to reach the ribosome, their intracellular sites of action, with high efficiency. In contrast, unencapsulated saporin could not be internalized on its own and resulted in negligible cytotoxicity even at high concentrations. Our data demonstrate that C5 end-capped branched PBAEs are a versatile and robust protein delivery platform, enabling cytosolic, functional protein delivery to a variety of cell lines. The polymers are largely agnostic to the size and surface charge of the protein cargo that they carry (Fig. 4E and table S1), unlike traditional PBAEs that depend on electrostatic interactions and can only encapsulate strongly negatively charged cargos such as nucleic acids.

### CRISPR gene editing through RNP delivery in vitro

**Fig. 5:**
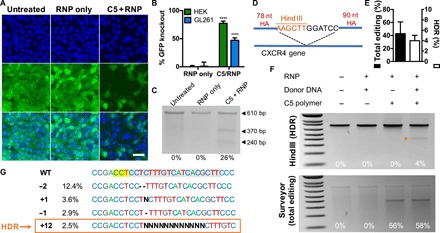
C5 nanoparticle delivery of Cas9 RNPs enables robust CRISPR gene editing in vitro. (**A**) Fluorescence microscopy images of HEK-GFPd2 cells treated with RNPs alone or C5 + RNPs; C5 + RNPs enabled knockout of GFP fluorescence. Scale bar, 50 μm. (**B**) Flow cytometry quantification of GFP knockout in HEK and GL261 cells. Data are means + SD (*n* = 4). Editing level of the C5/RNP group for each cell line was compared to that of the corresponding RNP-only group using Holm-Sidak corrected multiple *t* tests. ****P < 0.0001. (**C**) Surveyor mutation detector assay of GL261-GFPd2 cells treated with C5 + RNP nanoparticles. (**D**) Experimental design of HDR assay in the *CXCR4* gene; knock-in of a 12-nt insert flanked by homology arms (HA) results in the addition of a Hind III restriction enzyme site. (**E**) Quantification of total editing (via Surveyor assay) and HDR (via Hind III restriction digest) in HEK cells. Data are means + SD (*n* = 3). (**F**) Hind III restriction enzyme assay (top) and Surveyor assay (bottom) of HEK cells treated with different C5/RNP/donor DNA combinations; the orange arrowhead indicates HDR. (**G**) Inference of CRISPR Edits analysis of Sanger sequencing data from C5 + RNP + donor DNA-treated cells provides a breakdown of different edits. Percentages indicate the percentage of the total DNA population with the indicated genotype. The targeted sequence is highlighted in gray, and the protospacer adjacent motif sequence is highlighted in yellow.

C5 polymers were also used to encapsulate and deliver Cas9 RNPs to enable CRISPR gene editing in vitro. In these experiments, Cas9 protein and gene-targeting short guide RNA (sgRNA) were first incubated together at room temperature for 10 min to allow RNP self-assembly and then simply mixed with polymers to form nanoparticles. Delivery of RNPs targeting the GFP gene in cells constitutively expressing the GFP reporter resulted in 77% GFP knockout in HEK cells and 47% GFP knockout in GL261 murine glioma cells, as quantified by flow cytometry (Fig. 5B). Surveyor mutation detection assay was also performed to verify that loss in GFP fluorescence was due to perturbations in genomic DNA (Fig. 5C). RNPs were membrane impermeable on their own, and treatment with RNP alone yielded negligible levels of gene editing.

Next, we investigated the capability of C5/RNP nanoparticles to edit an endogenous gene through homology-directed repair (HDR). Cas9 protein was first self-assembled into RNPs with sgRNA targeting the human *CXCR4* gene, and RNPs were further mixed with a single-stranded DNA (ssDNA) repair template before mixing with C5 polymer. The ssDNA repair template included ~80 nucleotide (nt) homology arms flanking a 12 nt insert containing a Hind III restriction enzyme site (Fig. 5D). Successful HDR was quantified by Hind III restriction digest of polymerase chain reaction (PCR) amplicons of the genomic *CXCR4* site, while total amount of editing [nonhomologous end joining (NHEJ) and HDR] was quantified using the Surveyor mutation detection assay. Our results indicate that C5 nanoparticles successfully delivered the combination of RNP + ssDNA into HEK-293T cells. Gel electrophoresis analysis of cleavage products indicate that 4% HDR was achieved, while over 50% total editing was achieved (Fig. 5, E and F). Inclusion of an ssDNA template into the nanoparticle self-assembly process did not change the total level of editing achieved. Inference of CRISPR Edits (*21*) analysis of Sanger sequencing results confirmed the presence of a 12-bp insert in 2.5% of DNA sequences (Fig. 5G).

### Validation of CRISPR-stop reporter system to assess gene deletion

**Fig. 6:**
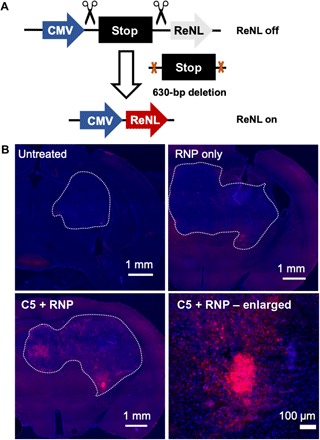
C5/RNP nanoparticles enable CRISPR editing in vivo. (**A**) Schematic of CRISPR-stop gene construct; deletion of a 630-bp expression stop cassette turns on downstream ReNL expression. (**B**) Direct intracranial administration of C5/RNP nanoparticles to an orthotopic GL261-stop-ReNL tumor enabled CRISPR editing in vivo. Nanoparticles were formulated at 3.5-pmol RNP with C5 polymer (15 w/w). Tumor boundary is outlined in white.

We engineered a CRISPR-stop reporter construct wherein a 630-bp stop-of-transcription cassette is placed upstream of a red-enhanced nanolantern (ReNL) fluorescent reporter (Fig. 6A). This CRISPR-stop construct was integrated into the genomic DNA of GL261 and B16-F10 cells via a PiggyBac transposon, and targeting CRISPR RNPs to regions flanking the stop cassette resulted in deletion of the stop cassette and turning on of ReNL fluorescence. We chose this system to evaluate in vivo gene editing as gain-of-function ReNL expression via dual-cut gene deletion could be easily and clearly detected.

In vitro assessment of this CRISPR-stop system using C5/RNP nanoparticles indicated that 16 and 43% editing were achieved in GL261 and B16 cells, respectively (fig. S6A). Compared to commercially available CRISPR delivery agents, C5/RNP nanoparticles enabled significantly higher editing levels than Lipofectamine CRISPRMax at all RNP doses tested and significantly higher editing levels than jetCRISPR at equimolar RNP doses tested (fig. S6B). jetCRISPR enabled significantly higher levels of editing than C5/RNP nanoparticles only when twice the RNP dose was used, further demonstrating the utility of the C5/RNP nanoparticle system in delivering CRISPR RNPs.

This reporter system also allowed us to easily assess the stability of our nanoparticles under physiological conditions. We preincubated C5/RNP nanoparticles in serum-containing complete cell culture media at 37°C for up to 4 hours before adding to cells and assessed their ability to induce gain-of-function CRISPR-stop edits (fig. S7A). Flow cytometry data revealed that no significant loss of nanoparticle efficacy was observed until preincubation time reached 4 hours, at which time delivery efficacy dropped by 25%. This is likely due to PBAE hydrolysis and is consistent with previous reports of PBAE half-life in aqueous conditions of 4 to 6 hours, a benefit to facilitate fast biodegradation and minimized toxicity in vivo (*22*). To achieve greater long-term stability of nanoparticles, as would be required for storage and supply chain management, we demonstrated that C5/RNP nanoparticles retain their efficacy following lyophilization with sucrose as a cryoprotectant, which may be the first documented case of a functional lyophilized RNP formulation (fig. S7B) (*23*).

### CRISPR editing in murine glioma tumors in vivo

Last, we investigated the ability of C5/RNP nanoparticles to enable CRISPR gene editing in vivo. In vivo assessment of gene editing was performed following intracranial implantation of GL261 cells constitutively expressing the CRISPR-stop construct into C57BL/6J mice. C5/RNP nanoparticles were infused intracranially through convection-enhanced delivery (CED) 10 days after tumor inoculation, and mice were euthanized and brains were extracted 6 days after nanoparticle CED. Histological analysis of mouse brains treated with C5/RNP nanoparticles (3.5-pmol RNP dose with 15 w/w polymer) revealed bright ReNL fluorescence within the tumor bulk, which was not observed in mice that received naked RNP infusion only (Fig. 6B and fig. S8). Although the brightest ReNL signal was localized in closest proximity to the injection site, ReNL expression could be detected several millimeters away from the primary injection site. These results demonstrate proof of principle that C5/RNP nanoparticles can enable localized CRISPR gene editing in vivo.

## DISCUSSION

Functional cytosolic protein delivery holds great value in biological research as well as therapeutic applications by enabling the perturbation of intracellular pathways previously undruggable by small-molecule drugs. To overcome the intrinsic cell membrane impermeability of many proteins, we synthesized and validated a series of carboxylated branched PBAEs for the encapsulation of a variety of different protein types into self-assembled nanoparticles. Carboxylate ligand chain length and hydrophobicity played an important role in polymer-protein interactions, as well as the ability for protein-encapsulated nanoparticles to interact with cellular and endosomal membranes. Polymers terminated with C5, a carboxylate ligand of moderate hydrophobicity, outperformed other ligands in the series both in the level of cellular internalization and endosomal disruption. The superior performance of C5 over end caps of lesser hydrophobicity could be explained by the fact that increased hydrophobicity facilitates nanoparticle stabilization through hydrophobic effects. Furthermore, the hydrocarbon chains in the polymer end-group could also interact with membranes, facilitating cellular internalization as well as endosomal escape through transient membrane perturbations. A similar phenomenon has been extensively reported with lipid-like materials and may also be applicable here (*24*). On the other hand, polymer end-groups such as C10 may be too hydrophobic, or else allow too long of a linker length, to efficiently interact with proteins. For example, a potential collapse of the hydrocarbon tail in aqueous buffer could obstruct interactions between the carboxylic acid functional group with proteins and cell membranes. This biphasic response is consistent with that reported by Ayala *et al.* (*25*) when similar amino acid analogs were used for hydrogel synthesis.

We further demonstrated the robustness of our nanoparticle system by cytosolically delivering a variety of proteins of different size and surface charge. The ability to functionally deliver the ribosome-inactivating protein saporin, which has an isoelectric point of 9.5 (*20*) and is thus strongly cationic at the pH of nanoparticle formation, validates our hypothesis that carboxylated PBAEs can rely on interactions beyond purely electrostatic forces to complex protein cargo into nanoparticles. This is a significant innovation upon traditional gene delivery PBAEs engineered to complex only highly anionic nucleic acid cargos through charge interactions (*26*).

Last, we demonstrated that C5 polymers were capable of functional delivery of Cas9 RNPs to enable CRISPR gene editing. In vitro delivery of RNPs targeting a GFP reporter gene resulted in nearly 80% GFP knockout following NHEJ. This level of gene knockout is comparable to that achieved by the DNA nanoclew system developed by Sun *et al*. (*27*) and significantly higher than that reported by Alsaiari *et al.* (*13*) using ZIF-8 metal-organic framework nanoparticles for CRISPR RNP delivery. Compared to commercially available CRISPR delivery reagents Lipofectamine CRISPRMax and jetCRISPR, C5/RNP nanoparticles enabled significantly higher levels of gene editing at manufacturer-recommended RNP doses. Furthermore, co-delivery of RNPs targeting the human *CXCR4* gene and an ssDNA repair template in the same self-assembled nanoparticle enabled 4% HDR in HEK-293T cells, which is significantly higher than that achieved by the CRISPR-Gold system developed by Lee *et al*. (*28*) in the same cell line when scaled by the RNP dose delivered. For translation considerations, the C5/RNP + ssDNA–encapsulated nanoparticles can be formulated by simply mixing with polymers, while the aforementioned CRISPR-Gold requires a multistep synthesis scheme including covalent conjugation of DNA sequences.

C5/RNP nanoparticles induced CRISPR gene editing in vivo as well using a challenging reporter model requiring a 631-bp deletion for gain-of-function fluorescence. In a proof-of-principle study, we demonstrated that deletion of an expression stop cassette resulted in gain-of-function ReNL reporter fluorescence upon intracranial injection of C5/RNP nanoparticles in a mouse glioma model. The highest levels of editing occurred near the primary nanoparticle infusion site, covering a region in the brain approximately 0.4 mm^2^ in area, which is comparable to that reported by Wang *et al.* (*9*), who used bioreducible lipids to deliver 5-pmol supercharged GFP-Cre to mouse brains containing a CRISPR-stop reporter system in which the stop cassette is flanked by LoxP sites. In another study, Staahl *et al*. (*29*) used a protein engineering approach to enable cellular internalization by adding 4× nuclear localization signal (NLS) residues to the N terminus of the Cas9 protein but required an order of magnitude higher RNP dose to achieve widespread editing. Intracranial injection of 4-pmol modified RNPs enabled gain-of-function tdTomato fluorescence in mouse brain regions similar in area to that observed by Wang *et al*. (*9*). In comparing in vivo editing efficiency, it is important to note that gene editing occurred in primary mouse neurons in the two abovementioned studies, while our study investigated gene editing in orthotopic mouse brain tumors. However, the bright ReNL signal induced by the C5/RNP nanoparticles highlights their robust in vivo delivery capabilities.

A putative advantage of this polymeric nanoparticle–based protein delivery system is its potential ability to evade immune responses. It has been demonstrated that PBAE nanoparticles optimized for nucleic acid delivery could be administered repeatedly to immunocompetent animals without a reduction in transfection efficacy (*30*), indicating that neutralizing antibodies were not formed against the nanoparticles. We hypothesize that our related nonviral protein delivery system could have similarly low levels of vector-mediated immune responses, which may be a significant advantage over traditional viral delivery vectors for which immunogenicity is a serious concern. Immunogenicity to Cas9 protein cargo may be a concern for direct in vivo CRISPR editing in human patients as Charlesworth *et al.* (*31*) recently reported that preexisting immunity against spCas9 is likely to limit the editing efficacy of CRISPR RNPs delivered to human patients. Polymeric nanoparticle encapsulation may attenuate immune responses against the protein cargo itself by protecting against circulating neutralizing antibodies, enabling CRISPR gene editing in patients with preexisting immunity. This effect was not evaluated in the current study but would be an interesting direction for future investigation.

In summary, we have reported here a polymeric nanoparticle system that can encapsulate and enable robust cytosolic delivery of a variety of different protein types, including potent cytotoxic agents as well as CRISPR-Cas9 RNPs. RNP delivery in vitro and in vivo induced high levels of gene editing at relatively low RNP doses. Biodegradable nanoparticles were formulated via a facile, highly scalable self-assembly process that is also amenable to lyophilization and storage. This versatile protein delivery platform provides a powerful tool for biological research as well as potential therapeutic applications for neurological disorders and beyond.

## MATERIALS AND METHODS

### Materials

Acryloyl chloride (CAS 814686), glycine (CAS 56-40-6), 4-aminobutanoic acid (CAS 56-12-2), 6-aminocaproic acid (CAS 60-32-2), 8-aminooctanoic acid (CAS 1002-57-9), 11-aminoundecanoic acid (CAS 2432-99-7), B7 (CAS 4687949), B8 (CAS 15625895), E1 (CAS 109-76-2), FITC-BSA, saporin from *Salvia officinalis* seeds, FITC-IgG from human serum, and Cas9-NLS were purchased from Sigma-Aldrich (St. Louis, MO). S4 (CAS 133251005) was purchased from Alfa Aesar (Tewksbury, MA).

### Carboxylate ligand synthesis

Carboxylate ligands were synthesized according to the method by Ayala *et al*. (*25*). Briefly, 0.1-mol carboxylate precursor molecule (listed in fig. S2B) was added at a 1:1.1 molar ratio with NaOH and dissolved in 80 ml of deionized water with vigorous stirring in an ice bath. Acryloyl chloride (0.11 mol) in 15 ml of tetrahydrofuran was added dropwise, and the pH of the reaction was maintained at 7.5 to 7.8 with 1 M NaOH solution. The reaction was allowed to proceed overnight before being acidified to the pH listed in fig. S2B with 1 M HCl solution and extracted three times with ethyl acetate. The organic layer was collected and dried with sodium sulfate, and the solvent was removed with rotary evaporation to yield a white powder.

### Polymer synthesis

Monomers B7 and B8 were dissolved in anhydrous dimethyl sulfoxide (DMSO) at 0.8:0.2 molar ratio, and monomer S4 was added at an overall vinyl/amine ratio of 2.2:1 to a final monomer concentration of 150 mg/ml. The reaction was allowed to proceed at 90°C with stirring overnight, at which point polymers were end-capped by reacting with monomer E1 (0.2 M final concentration in DMSO) at room temperature for 2 hours. The resulting E1 polymers were purified by two diethyl ether washes, after which polymers were dissolved at 200 mg/ml in DMSO and end-capped with carboxylate ligands (0.2 M final concentration in DMSO) at room temperature for 2 hours. The resulting carboxylated polymers were further purified by ether precipitation, and the remaining solvent was removed in a vacuum chamber. Polymers were dissolved in DMSO at 100 mg/ml and stored in single-use aliquots at −20°C with desiccant.

### Polymer characterization: NMR, GPC, and pH titration

Polymer structure was characterized by nuclear magnetic resonance (NMR) spectroscopy via ^1^H NMR in CDCl_3_ (Bruker 500 MHz) and analyzed using TopSpin 3.5 software. Polymer molecular weight was characterized by gel permeation chromatography (GPC); polymers were dissolved in butylated hydroxytoluene–stabilized tetrahydrofuran with 5% DMSO and 1% piperidine, filtered through a 0.2-μm polytetrafluoroethylene filter, and characterized using GPC against linear polystyrene standards (Waters, Milford, MA). pH titrations were performed using a SevenEasy pH meter (Mettler Toledo) with 10 mg of polymer dissolved in 10 ml of 100 mM NaCl acidified with HCl, as previously described (*15*). Polymer was titrated from pH 3.0 to 11.0 using 100 mM NaOH added stepwise, and pH was recorded after each addition.

### Nanoparticle characterization

Nanoparticles were prepared by dissolving polymer and protein separately in 25 mM sodium acetate (NaAc; pH 5), mixing the two solutions at a 1:1 volume ratio, and allowing for nanoparticle self-assembly at room temperature for 10 min. To prepare nanoparticles encapsulating CRISPR RNPs, sgRNA and Cas9 protein were first mixed together at a 2:1 molar ratio to allow RNP assembly at room temperature for 10 min; RNPs were then mixed with polymers at a 1:1 volume ratio. Nanoparticles were diluted 1:5 in 150 mM phosphate-buffered saline (PBS) to determine particle size and zeta potential in neutral, isotonic buffer. Hydrodynamic diameter was measured via dynamic light scattering on a Malvern Zetasizer Pro (Malvern Panalytical); zeta potential was measured via electrophoretic light scattering on the same instrument. Transmission electron microscopy (TEM) images were acquired with a Philips CM120 (Philips Research). Nanoparticles encapsulating BSA (30 w/w) were prepared at a polymer concentration of 1.8 mg/ml in 25 mM NaAc. Thirty-microliter nanoparticles were added to 400–square mesh carbon-coated TEM grids and allowed to adhere for 20 min. Grids were then rinsed with ultrapure water and allowed to fully dry before imaging.

### sgRNA in vitro transcription

In vitro transcription was performed using a MEGAshortscript T7 Transcription Kit (Invitrogen) and purified using a MEGAclear Transcription Clean-Up Kit (Invitrogen) following the manufacturer’s instructions. The DNA templates used for in vitro transcription were synthesized as gBlocks from IDT (sequences are listed in table S2).

### Cell culture and cell line preparation

HEK-293T cells, GL261 murine glioma cells, CT-2A murine glioma cells, B16-F10 murine melanoma cells, and MSC-083 human primary adipose–derived mesenchymal stem cells were cultured in Dulbecco’s modified Eagle’s medium (Thermo Fisher Scientific) supplemented with 10% fetal bovine serum and 1% penicillin/streptomycin. To generate reporter cell lines for CRISPR editing experiments, HEK-293T and GL261 cells were induced to constitutively express a destabilized form of GFP (GFPd2) via a PiggyBac transposon/transposase system, as detailed previously (*16*). Similarly, GL261 and B16-F10 cells were induced to constitutively express a construct where transcription of a ReNL reporter gene is prevented by a dual-SV40 transcription stop cassette (CRISPR-stop). The PiggyBac transposon plasmids used to generate GFPd2^+^ and CRISPR-stop^+^ cell lines are available on Addgene as plasmid nos. 115665 and 113965, respectively.

### Transfection

Cells were plated at a density of 15,000 cells per well in 96-well tissue culture plates and allowed to adhere overnight. Protein-encapsulated nanoparticles were prepared as described above, and optimal nanoparticle formulations for each protein are listed in table S1. Twenty-microliter nanoparticles were added per well into serum-containing complete cell culture media and incubated with cells for 4 hours. For FITC-BSA uptake experiments, the nanoparticle/media mixture was removed at 4 hours, cells were washed three times with PBS, and uptake was assessed via flow cytometry using a BD Accuri C6 flow cytometer (BD Biosciences). Nanoparticle uptake was quantified by normalizing the geometric mean fluorescence of treated wells to that of untransfected controls.

For all other transfection experiments, fresh complete medium was replenished after 4 hours of incubation with nanoparticles. For saporin transfection experiments, cell killing was assessed 2 days after transfection. Cells were stained with Hoechst 33342 nuclear stain (1:5000 dilution) and propidium iodide (1:500 dilution) for 20 min and imaged and analyzed using Cellomics ArrayScan VTI with live cell imaging module (Thermo Fisher Scientific). Cell killing was calculated by normalizing live cell numbers in wells treated with C5/saporin nanoparticles to those in wells treated with matching nanoparticle formulations delivering nontoxic BSA. For CRISPR RNP transfection experiments, gene editing was assessed 3 days after transfection. GFPd2 knockout and turning on of ReNL were assessed via flow cytometry. GFPd2 knockout was quantified by normalizing the GFP geometric mean fluorescence of C5/RNP-treated wells to that of untransfected control wells; gain of ReNL fluorescence was quantified as the percentage of cells positively expressing ReNL when gated against untreated control.

For CRISPR HDR experiments, Cas9 and sgRNA targeting the *CXCR4* gene were first mixed at a 1:2 molar ratio and allowed to self-assemble into RNPs. The ssDNA repair template was then added at a 1:1 volume ratio to the RNPs, and the combined solution was mixed with C5 polymer to allow for nanoparticle self-assembly. Each well received a final dose of 300 ng of sgRNA, 690 ng of Cas9 protein, and 400 ng of ssDNA repair template.

### Commercial reagent RNP delivery

For CRISPR RNP delivery experiments using commercial reagents, B16-F10 cells expressing the PiggyBac CRISPR-stop^+^ cassette were plated in 96-well plates 24 hours prior. Lipofectamine CRISPRMax and jetCRISPR commercial reagents designed for RNP delivery were formulated with SpCas9 RNP nanoparticles according to the manufacturer’s instructions and added to cells at the specified doses. Specifically, RNPs were formulated as recommended at 1:1 molar ratio of SpCas9 to sgRNA using the single-guide CRISPR-stop sgRNA and complexed with commercial reagents before adding to cells and incubating 48 hours. Editing efficacy was assessed 2 days following RNP delivery using flow cytometry to assess percentage of cells expressing ReNL from the 630-bp deletion of the CRISPR-stop^+^ cassette.

### Endocytosis pathway inhibition

HEK-293T cells were plated for transfection as described above and incubated for 1 hour with endocytosis inhibitors (*32*) in complete cell culture media immediately before transfection. Chlorpromazine (3 μg/ml) was used to inhibit clathrin-mediated endocytosis; methyl-β-cyclodextrin (7.5 mg/ml) was used to inhibit lipid raft–mediated endocytosis; genistein (10 μg/ml) was used to inhibit caveolin-mediated endocytosis; cytochalasin D (0.5 μg/ml) was used to inhibit actin polymerization and macropinocytosis. C5/FITC-BSA nanoparticles were formulated at 300 ng of protein per well and 30 w/w. Nanoparticles were incubated with cells for 2 hours, at which time they were washed with PBS and analyzed via flow cytometry to assess nanoparticle uptake. Endocytosis inhibition was quantified by normalizing the geometric mean fluorescence of wells treated with inhibitor to that of untransfected control wells.

### Gal8-GFP recruitment assay

The Gal8-GFP recruitment assay to assess endosomal disruption was based on methods previously reported by Kilchrist *et al*. (*18*). Briefly, B16-F10 cells were made to constitutively express a Gal8-GFP fusion protein using a PiggyBac transposon plasmid (127191, Addgene). Nanoparticles encapsulating BSA (125 ng of BSA per well, 25 w/w) were incubated with cells for 4 hours, at which point cells were replenished with complete media and stained with Hoechst 33342 nuclear stain (1:5000 dilution). Gal8-GFP recruitment was imaged and analyzed with Cellomics ArrayScan VTI with live cell imaging module; cell count was generated using an algorithm to extrapolate area surrounding Hoechst-stained cell nuclei, and endosomal disruption was reported as the average number of punctate Gal8-GFP spots per cell.

### Cellular viability

Cell viability was assessed 24 hours after transfection using MTS CellTiter 96 Aqueous One Solution Cell Proliferation Assay (Promega) following the manufacturer’s instructions. Cell viability of treated cells was normalized to that of untreated controls (*n* = 4 ± SEM).

### Confocal microscopy

HEK-293T or MSC-083 cells were plated on Nunc Lab-Tek 8 chambered borosilicate coverglass well plates (155411, Thermo Fisher Scientific) at 30,000 cells per well in 250 μl of phenol red–free Dulbecco’s modified Eagle’s medium supplemented with 10% fetal bovine serum and 1% penicillin/streptomycin 1 day before transfection. C5 nanoparticles were prepared at 30 w/w with the indicated proteins, and 50 μl of nanoparticles was administered per well for a total dose of 300 ng of protein. Nanoparticles were incubated with cells for 4 hours, at which time cells were replenished with fresh complete medium and stained with Hoechst 33342 nuclear stain at a 1:5000 dilution and Cell Navigator Lysosome Staining dye (AAT Bioquest). Excess stain was washed away, and cells were imaged in live cell imaging solution at 37°C in 5% CO_2_. Images were acquired at Nyquist limit resolution using a Zeiss LSM 780 microscope with Zen Blue software and 63× oil immersion lens. Specific laser channels used were 405-nm diode, 488-nm argon, 561-nm solid-state, and 639-nm diode lasers. Laser intensity and detector gain settings were maintained across all image acquisition for each experiment.

### Indel quantification via Surveyor assay

Genomic DNA from cells treated with C5/RNP nanoparticles and untransfected controls were isolated using a GeneJET Genomic DNA Purification Kit (Thermo Fisher Scientific). A 660-bp region flanking the predicted cut site was PCR-amplified (primers are listed in table S2), and PCR products were purified using a QIAquick PCR Purification Kit (Qiagen). Four hundred nanograms of PCR amplicons was hybridized in the presence of 50 mM KCl, and the Surveyor mutation detection assay (IDT) was performed following the manufacturer’s instructions. The DNA products were then run on a 2% agarose gel stained with ethidium bromide in TBE (tris-borate-EDTA) buffer (80 V for 50 min) and imaged under ultraviolet light. DNA band intensity was quantified using ImageJ image analysis software, and indel rate was calculated on the basis of the method by Schumann *et al*. (*33*).

### HDR quantification via restriction enzyme digest

A restriction enzyme–based assay to quantify HDR rates was adapted from methods reported by Lee *et al*. (*28*). Briefly, an HDR repair template was designed to insert a 12-nt region that includes the Hind III restriction site into the *CXCR4* gene, with 78-nt homology arm upstream and 90-nt homology arm downstream of the insert site. The repair template was synthesized as an ssDNA oligo from IDT (sequence is listed in table S2). Genomic DNA of cells treated with C5/RNP + ssDNA nanoparticles or control nanoparticles was harvested 5 days after transfection. A 770-bp region surrounding the edit site was PCR-amplified, and the PCR amplicon was digested with Hind III (0.01 enzyme units/ng DNA) for 1 hour at 37°C before standard gel electrophoresis as described above. Percent HDR was calculated by dividing the band intensity of the digested fragment (approximately 400 bp) by the band intensity of all bands in the lane.

### GL261-CRISPR-stop tumor implantation

All animal work was done in strict adherence to the policies and guidelines of the Johns Hopkins University Animal Care and Use Committee. For intracranial tumor implantations, 6- to 8-week-old female C57BL/6J mice (the Jackson Laboratory) were anesthetized using a ketamine (100 mg/kg)/xylazine (10 mg/kg) cocktail (10 mg/kg) and mounted on a stereotaxic frame. A rostrocaudal incision was made with a scalpel, the surface of the skull was exposed and cleaned with 100% ethanol, and a small burr hole was made in the skull 4 mm posterior to the coronal suture and 2 mm lateral to the sagittal suture using an electric drill. A total of 130,000 GL261 murine glioma cells engineered to constitutively express the CRISPR-stop construct were implanted into mouse brain parenchyma through the burr hole using a 10-μl Hamilton syringe (Hamilton Company); the injection volume was 2 μl.

### Intratumoral C5/RNP nanoparticle injection

Tumors were allowed to form for 10 days, at which time C5/RNP nanoparticle administration began. Nanoparticles were formed in PBS buffer at a final polymer concentration of 0.86 mg/ml and 3.5-pmol RNPs (15 w/w) immediately before injection. Mice were anesthetized with a ketamine cocktail (10 mg/kg) as described earlier, and the original incision was opened. CED was performed using a 26-gauge Hamilton needle stereotaxically placed at a depth of 3 mm, and an UltraMicroPump (UMP3) with SYS-Micro4 Controller (World Precision Instruments, Sarasota, FL) was used to infuse nanoparticles at a rate of 0.5 μl/min (*34*). Ten microliters of nanoparticles was injected per animal, after which the needle was left at the injection site for 5 min to mitigate backflow. Following needle removal, the incision was closed with 4-0 silk sutures, and the animal was allowed to awaken and recover.

### In vivo visualization of ReNL reporter

For ReNL reporter analysis, 6 days after injection, mice were anesthetized and perfused with 4% paraformaldehyde. Brains were extracted, postfixed overnight, and soaked in 30% sucrose for 24 hours. Brains were frozen on dry ice and mounted onto a cryostat sample holder using Optimal Cutting Temperature compound, cryosectioned (coronal plane sections) using a Leica CM 3050 S cryostat (Leica Biosystems), and the prepared 40-μm sections were mounted onto glass slides with Hoechst nuclear stain (1:4000 dilution) and SlowFade Gold Antifade Reagent (Thermo Fisher Scientific). Mounted sections were stored at −80°C and protected from light until use. Sections were imaged by fluorescence microscopy using a Zeiss Apotome.2 microscope with Zen Blue software. Microscope settings were maintained across all image acquisition.

### Nanoparticle stability

To characterize nanoparticle stability over time under physiological conditions, C5/RNP nanoparticles were incubated in serum-containing complete cell culture medium at 37°C and added to GL261-CRISPR-stop cells at designated time points up to 4 hours. C5/RNP nanoparticles were also lyophilized with sucrose (30 mg/ml) as cryoprotectant following previously reported protocols (*35*) and stored at −20°C for 4 days before adding to cells. Cells were incubated with nanoparticles for 3 hours, and the level of gene editing was analyzed via flow cytometry 3 days after transfection.

### Statistics

Prism 6 (GraphPad, La Jolla, CA) was used for all statistical analyses and curve plotting. Statistical tests were performed with a global α value of 0.05. Unless otherwise stated, absence of statistical significance markings where a test was stated to have been performed signified no statistical significance. The statistical test used and the number of experimental replicates are listed in the captions for each figure. Statistical significance was denoted as follows: *P < 0.05, **P < 0.01, ***P < 0.001, and ****P < 0.0001.

## Supplementary Material

http://advances.sciencemag.org/cgi/content/full/5/12/eaay3255/DC1

Download PDF

Carboxylated branched poly(β-amino ester) nanoparticles enable robust cytosolic protein delivery and CRISPR-Cas9 gene editing

## SUPPLEMENTARY MATERIALS

Supplementary material for this article is available at http://advances.sciencemag.org/cgi/content/full/5/12/eaay3255/DC1 Fig. S1. Synthesis and characterization of carboxylated branched PBAE polymers. Fig. S2. Synthesis and characterization of carboxylate ligands. Fig. S3. Cell viability after treatment with carboxylated branched PBAE protein nanoparticles. Fig. S4. Confocal images of cells treated with C5/FITC-BSA nanoparticles. Fig. S5. Characterization of polymer pH buffering and endosomal disruption capabilities. Fig. S6. C5/RNP nanoparticles enable in vitro gene deletion. Fig. S7. C5/RNP nanoparticles are stable in serum-containing media and in lyophilized form. Fig. S8. C5/RNP nanoparticle-enabled in vivo CRISPR editing is reproducible. Table S1. Characteristics of proteins and encapsulated C5 nanoparticles and optimal nanoparticle formulations used in this study. Table S2. DNA sequences.
