# Supplementary material for: Carboxylated branched poly(β-amino ester) nanoparticles enable robust cytosolic protein delivery and CRISPR-Cas9 gene editing
Source: Sci Adv. 2019 Dec 6;5(12):eaay3255. doi: 10.1126/sciadv.aay3255 (PMC6897553; doi:10.1126/sciadv.aay3255)
Supplement: http://advances.sciencemag.org/cgi/content/full/5/12/eaay3255/DC1 [file supp_5_12_eaay3255__index.html]

Science Advances | Science AdvancesAAASSearchScience AdvancesMenu

## Supplementary Materials

**This PDF file includes:**

- Fig. S1. Synthesis and characterization of carboxylated branched PBAE polymers.
- Fig. S2. Synthesis and characterization of carboxylate ligands.
- Fig. S3. Cell viability after treatment with carboxylated branched PBAE protein nanoparticles.
- Fig. S4. Confocal images of cells treated with C5/FITC-BSA nanoparticles.
- Fig. S5. Characterization of polymer pH buffering and endosomal disruption capabilities.
- Fig. S6. C5/RNP nanoparticles enable in vitro gene deletion.
- Fig. S7. C5/RNP nanoparticles are stable in serum-containing media and in lyophilized form.
- Fig. S8. C5/RNP nanoparticle-enabled in vivo CRISPR editing is reproducible.
- Table S1. Characteristics of proteins and encapsulated C5 nanoparticles and optimal nanoparticle formulations used in this study.
- Table S2. DNA sequences.

Download PDF

**Files in this Data Supplement:**

- Adobe PDF - aay3255\_SM.pdf
