# Supplementary material for: Carboxylated branched poly(β-amino ester) nanoparticles enable robust cytosolic protein delivery and CRISPR-Cas9 gene editing
Source: Sci Adv. 2019 Dec 6;5(12):eaay3255. doi: 10.1126/sciadv.aay3255 (PMC6897553; doi:10.1126/sciadv.aay3255)
Supplement: Download PDF [file aay3255_SM.pdf]

Science Advances
AAAS

advances.sciencemag.org/cgi/content/full/5/12/eaay3255/DC1

Supplementary Materials for

Carboxylated branched poly(β-amino ester) nanoparticles enable robust cytosolic protein delivery and CRISPR-Cas9 gene editing

Yuan Rui, David R. Wilson, John Choi, Mahita Varanasi, Katie Sanders, Johan Karlsson, Michael Lim, Jordan J. Green*

*Corresponding author. Email: green@jhu.edu

Published 6 December 2019, *Sci. Adv.* **5**, eaay3255 (2019)
DOI: 10.1126/sciadv.aay3255

This PDF file includes:

Fig. S1. Synthesis and characterization of carboxylated branched PBAE polymers.
Fig. S2. Synthesis and characterization of carboxylate ligands.
Fig. S3. Cell viability after treatment with carboxylated branched PBAE protein nanoparticles.
Fig. S4. Confocal images of cells treated with C5/FITC-BSA nanoparticles.
Fig. S5. Characterization of polymer pH buffering and endosomal disruption capabilities.
Fig. S6. C5/RNP nanoparticles enable in vitro gene deletion.
Fig. S7. C5/RNP nanoparticles are stable in serum-containing media and in lyophilized form.
Fig. S8. C5/RNP nanoparticle-enabled in vivo CRISPR editing is reproducible.
Table S1. Characteristics of proteins and encapsulated C5 nanoparticles and optimal nanoparticle formulations used in this study.
Table S2. DNA sequences.

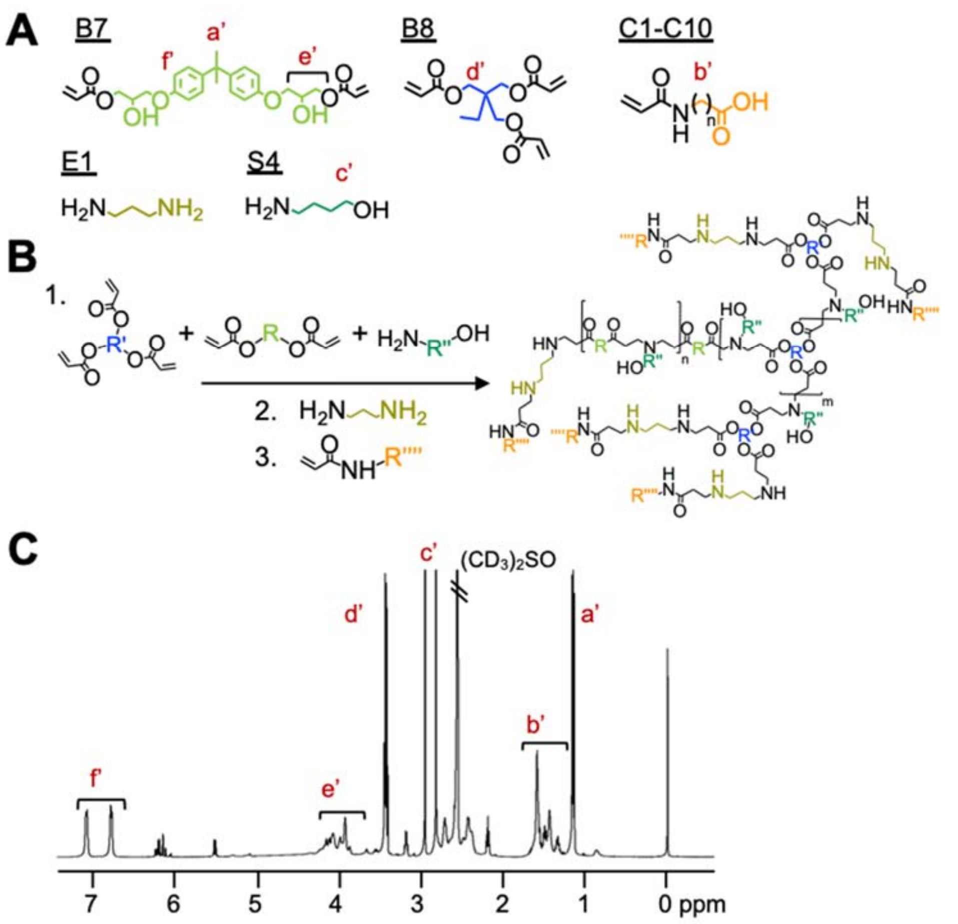

D

M_N	M_W	PDI
4275	10618	2.483

Fig. S1. Synthesis and characterization of carboxylated branched PBAE polymers. (**A**) Monomer structures. (**B**) Reaction scheme for branched polymers. (1) Acrylate-terminated branched PBAE is synthesized via Michael addition of B and S monomers; (2) polymer end-capping with monomer E1 results in amine-terminated polymers; (3) further end-capping with carboxylate ligands yields final polymer products. (**C**) ^{1}H NMR spectrum of polymer C5; distinctive peaks from each monomer are labeled according to chemical structures shown in (A). (**D**) Molecular weight data of polymer C5 obtained via GPC.

A

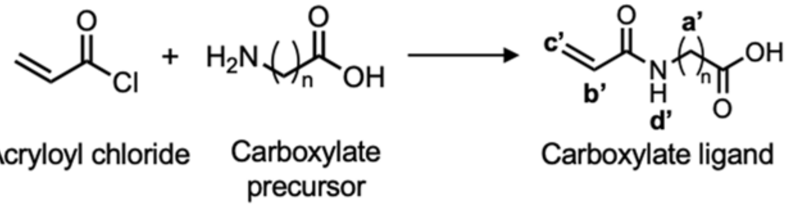

B

Ligand	Carboxylate precursor	Extraction pH
C1	Glycine	2.0
C3	4-aminobutyric acid	2.0
C5	6-aminocaproic acid	3.0
C7	8-amino-caprylic acid	5.0
C10	11-aminoundecanoic acid	5.0

C

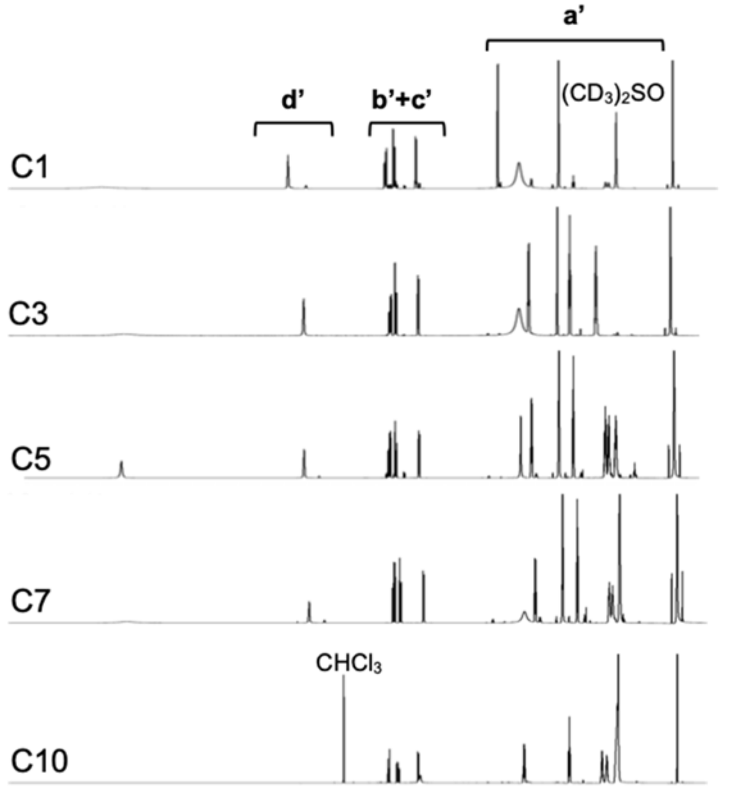

Fig. S2. Synthesis and characterization of carboxylate ligands. (**A**) Reaction route schematic. (**B**) Acidification pH for extraction of each ligand. (**C**) ^{1}H NMR spectrum of each ligand; peaks labeled according to the chemical structure shown in (A).

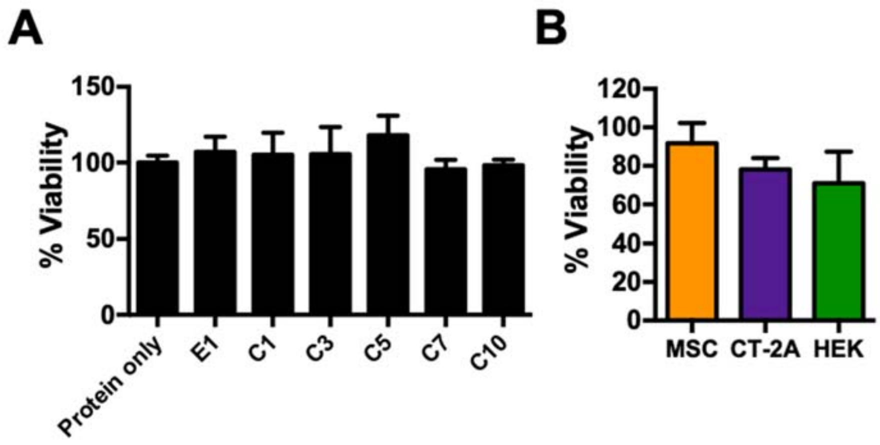

Fig. S3. Cell viability after treatment with carboxylated branched PBAE protein nanoparticles. (**A**) Cell viability of CT-2A murine glioma cells treated with E1-C10 nanoparticles encapsulating BSA. (**B**) Cell viability of other cell types treated with C5/BSA nanoparticles. Nanoparticle formulation used in both experiments is 300 ng protein per well at 20 w/w. Cell viability measured using MTT assay. Data presented as mean+SD; *n*=4.

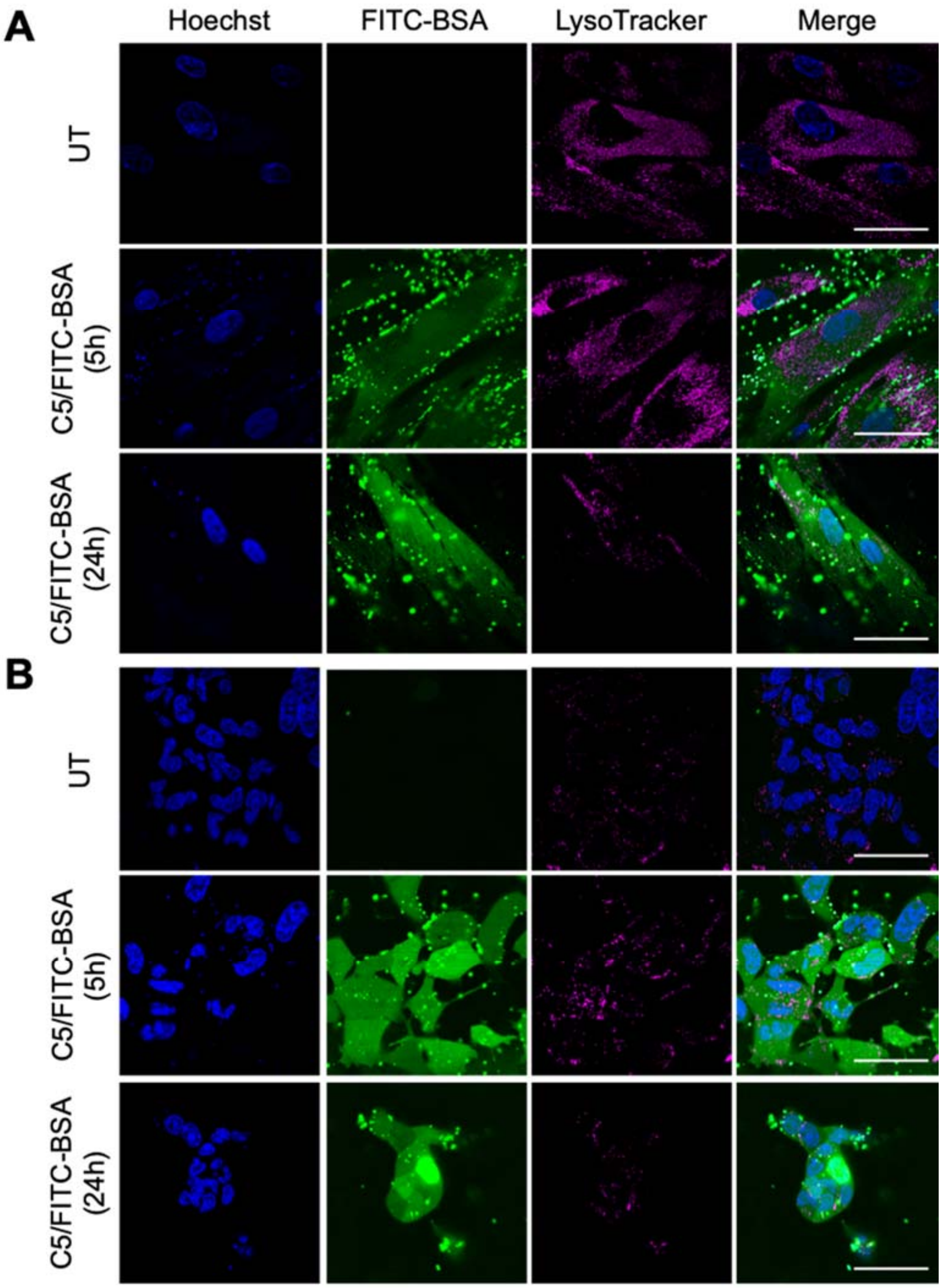

Fig. S4. Confocal images of cells treated with C5/FITC-BSA nanoparticles. (**A**) Human adipose-derived mesenchymal stem cells and (**B**) HEK-293T cells were treated with C5/FITC-BSA nanoparticles (300 ng protein dose, 30 w/w) and imaged at 5 and 24 hours post-transfection. Untreated cells (UT) were also imaged as controls. Scale bar = 10 µm.

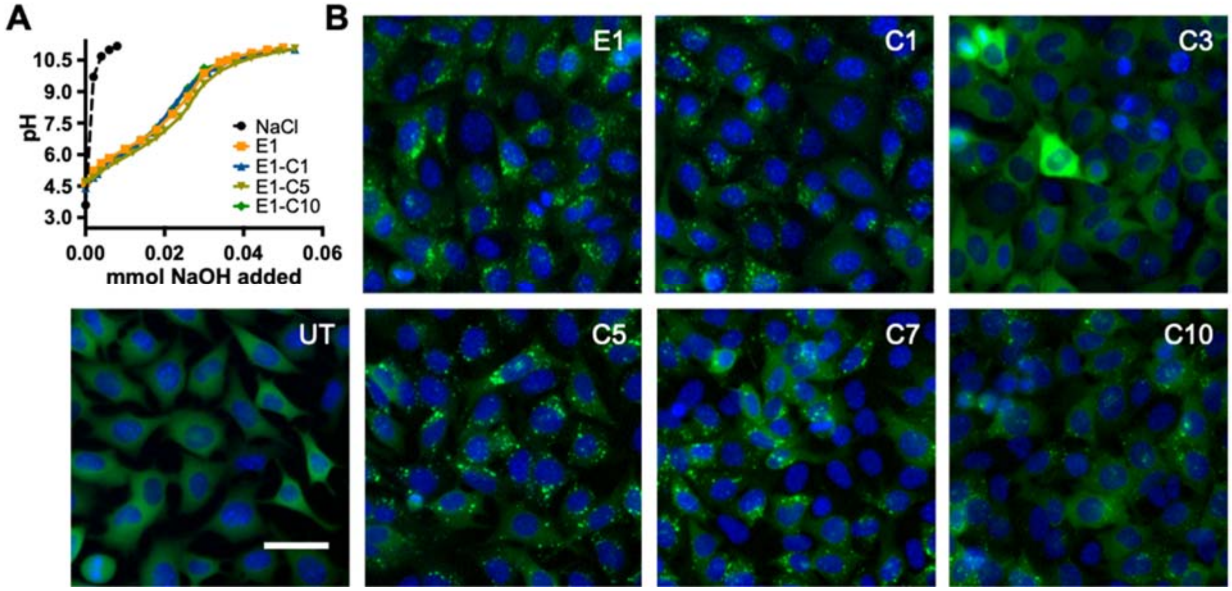

Fig. S5. Characterization of polymer pH buffering and endosomal disruption capabilities. (**A**) pH titration curve of several polymers in the series. Sigmoidal curve fit of the titration curves and comparison via sum-of-squares F-test statistically demonstrated that there was no significant difference between buffering in the range of pH 4.5-8 for carboxylate ligands (P = 0.062). (**B**) Representative images from high content imaging of B16-F10/Gal8-GFP cells treated with nanoparticles encapsulating BSA (125 ng BSA per well, 25 w/w; scale bar = 50 µm).

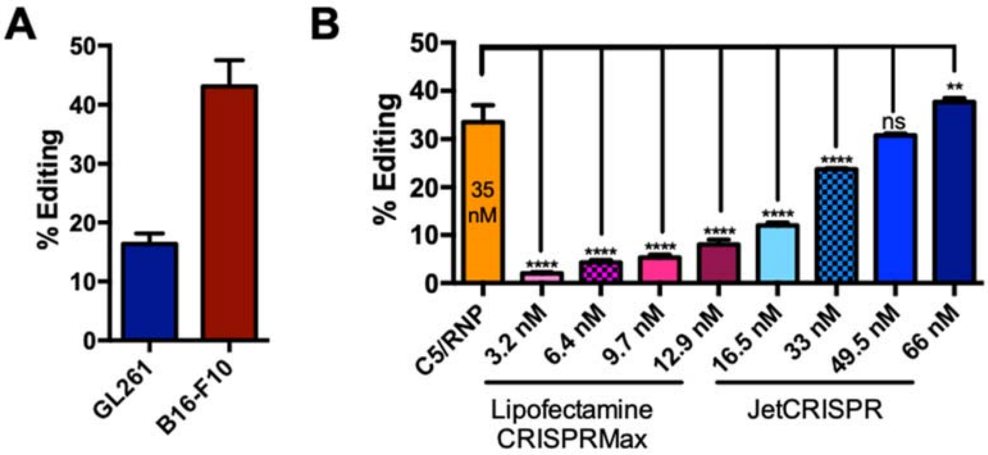

Fig. S6. C5/RNP nanoparticles enable in vitro gene deletion. (**A**) C5/RNP deletion of stop cassette *in vitro* in 2 murine cancer cell lines resulted in turning on of ReNL fluorescence as detected by flow cytometry. Data are mean+SD; *n*=4. (**B**) Comparison in gene editing performance with commercially-available CRISPR transfection reagents in B16-F10 cells. C5/RNP nanoparticles were administered at an RNP dose of 35 nM per well; RNP dose for commercial reagents are indicated. For each commercial reagent, the manufacturer recommended RNP dose is indicated by checkerboard pattern. Turning on of ReNL fluorescence was quantified by flow cytometry. Data are mean+SD; *n*=4. Statistical analysis performed using one-way ANOVA with Dunnett's post-hoc tests compared against the C5/RNP group; ***P*<0.01, *****P*<0.0001.

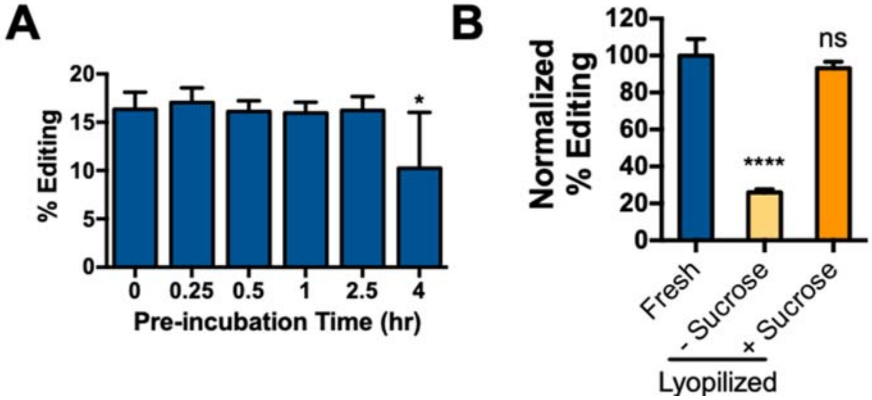

Fig. S7. C5/RNP nanoparticles are stable in serum-containing media and in lyophilized form. (**A**) % Editing observed in GL261-CRISPR-stop cells after treatment with nanoparticles pre-incubated in serum-containing complete medium at 37°C for the designated times. Statistical significance determined by one-way ANOVA with Dunnett's post-hoc tests as compared to time = 0 . Data presented as mean+SD; (n=4). *P<0.05. (**B**) Nanoparticles lyophilized with or without 30 mg/mL sucrose and stored at -20 °C prior to being added to cells. Percent editing normalized to fresh nanoparticles. Data presented as mean+SD; n=4. Statistical significance determined by one-way ANOVA with Dunnett's post-hoc tests as compared to fresh nanoparticles; (n=4). *P<0.05, ****P<0.0001.

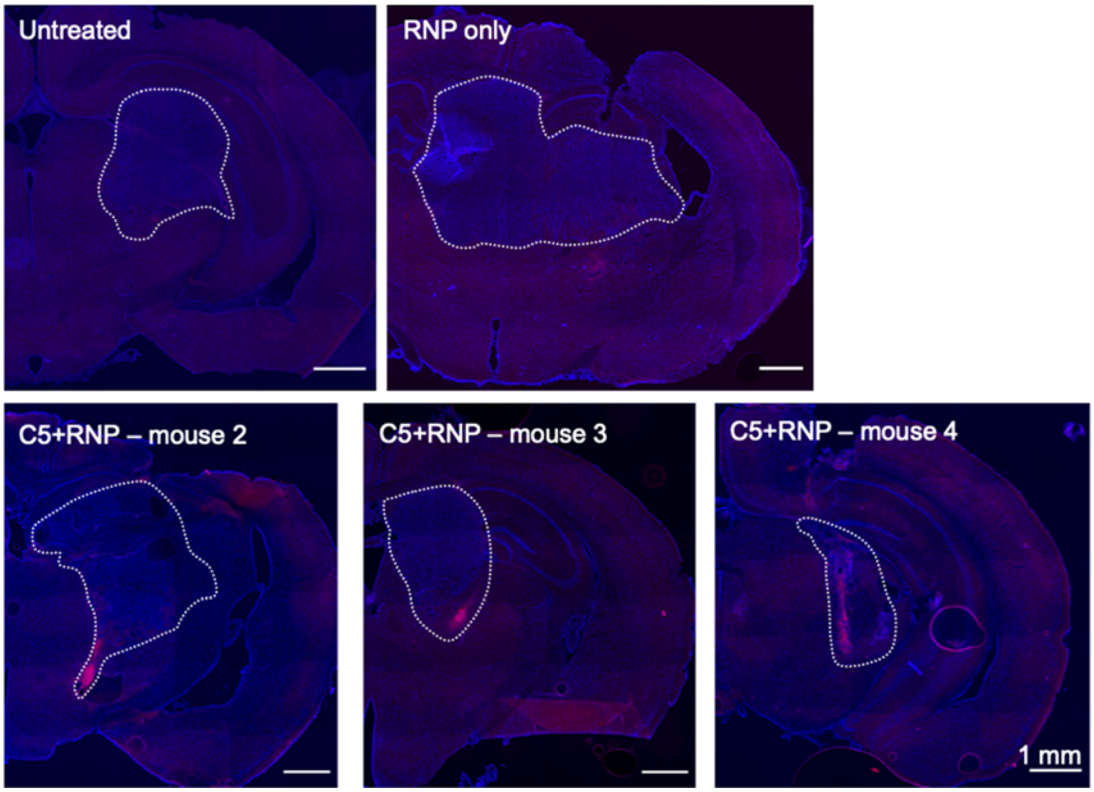

Fig. S8. C5/RNP nanoparticle-enabled in vivo CRISPR editing is reproducible. Red ReNL fluorescent signal indicating CRISPR editing can be detected in the 3 additional mice treated with C5/RNP nanoparticles while untreated and RNP only groups showed no signal. Nanoparticles were formulated at 3.5 pmol RNP with 15 w/w C5 polymer. Tumors boundary outlined in white.

Table S1. Characteristics of proteins and encapsulated C5 nanoparticles and optimal nanoparticle formulations used in this study.

Protein	Protein Characteristics		Nanoparticle Characteristics				
	MW	pI	Size (nm)	Zeta (mV)	Optimal Protein Dose	Optimal Polymer Dose (mg/mL)	Equivalent w/w
GFP	27 kD	5.8	150±50	9.9±0.7	300 ng	0.075	30
Saporin	29 kD	9.5	120±30	8.7±0.4	2.5-15 nM	0.075	2600-175
BSA	66.5 kD	4.7	160±60	5±1	300 ng	0.075	30
IgG	150 kD	6.6-7.2	120±20	-1±1	300 ng	0.075	30
Cas9	163 kD	9	180±10	12.3±0.2	690 ng	0.1	22

Table S2. DNA sequences.

		Sequences	Notes
Target Sequences	GFP	GGAGCGCACCATCTTCTTCAAGG	PAM; Positive strand
	CRISPR-stop	GTATAGCATACATTATACGAGG	PAM; Negative strand
	CXCR4	GAAGCGTGATGACAAAGAGGAGG	PAM; Negative strand
sgRNA IVT Template	GFP	*GTTTT*_TAATACGACTCACTATA_GGAGCGCACCATCTTCTTCAGTTTTAGAGCTAGAAATAGCAAGTTAAAATAAGGCTAGTCCGTTATCAACTTGAAAAAGTGGCACCGAGTCGGTGCTTTTTTT	_T7 promoter_<br>Target sequence<br>gRNA scaffold
	CRISPR-stop	*GTTTT*_TAATACGACTCACTATA_GGTATAGCATACATTATACGGTTTTAGAGCTAGAAATAGCAAGTTAAAATAAGGCTAGTCCGTTATCAACTTGAAAAAGTGGCACCGAGTCGGTGCTTTTTTT	
	CXCR4	*GTTTT*_TAATACGACTCACTATA_GAAGCGTGATGACAAAGAGGGTTTTAGAGCTAGAAATAGCAAGTTAAAATAAGGCTAGTCCGTTATCAACTTGAAAAAGTGGCACCGAGTCGGTGCTTTTTTT	
Primers for Surveyor® Assay	GFP_FWD	CTGGTCGAGCTGGACGGCGACG	Amplicon size: 630 bp
	GFP_REV	CACGAACTCCAGCAGGACCATG	
	CXCR4_FWD	TTAATTCTCTTGTGCCCTTAGCCCACTACTTCAG	Amplicon size: 770 bp
	CXCR4_REV	GGACAGGATGACAATACCAGGCAGGATAAGGCC	
HDR Donor Template	CXCR4	CCTGGTCATGGGTTACCAGAAGAAACTGAGAAGCATGACGGACAAGTACAGGCTGCACCTGTCAGTGGCCGACCTCCT_AAGCTTGGATCC_CTTTGTCATCACGCTTCCCTTCTGGGCAGTTGATGCCGTGGCAAACTGGTACTTTGGGAACTTCCTATGCAAGGCAGTCCATGTCATCTA	_Inserted region_<br>Homology arms<br>_HindIII restriction site_
